# Disordered Electron Transfer: New Forms of Defective Steroidogenesis and Mitochondriopathy

**DOI:** 10.1210/clinem/dgae815

**Published:** 2024-11-22

**Authors:** Walter L Miller, Amit V Pandey, Christa E Flück

**Affiliations:** Department of Pediatrics, Center for Reproductive Sciences, and Institute for Human Genetics, University of California, San Francisco, San Francisco, CA 94143, USA; Pediatric Endocrinology, Diabetology and Metabolism, Department of Pediatrics, Inselspital, Bern University Hospital, University of Bern, Bern 3010, Switzerland; Department of BioMedical Research, University of Bern, Bern 3010, Switzerland; Pediatric Endocrinology, Diabetology and Metabolism, Department of Pediatrics, Inselspital, Bern University Hospital, University of Bern, Bern 3010, Switzerland; Department of BioMedical Research, University of Bern, Bern 3010, Switzerland

**Keywords:** adrenal, cytochrome P450, electron transfer, ferredoxin, ferredoxin reductase, FDXR-related mitochondriopathy, mitochondria, mitochondrial neuropathy, oxidoreductase, congenital adrenal hyperplasia

## Abstract

Most disorders of steroidogenesis, such as forms of congenital adrenal hyperplasia (CAH) are caused by mutations in genes encoding the steroidogenic enzymes and are often recognized clinically by cortisol deficiency, hyper- or hypo-androgenism, and/or altered mineralocorticoid function. Most steroidogenic enzymes are forms of cytochrome P450. Most P450s, including several steroidogenic enzymes, are microsomal, requiring electron donation by P450 oxidoreductase (POR); however, several steroidogenic enzymes are mitochondrial P450s, requiring electron donation via ferredoxin reductase (FDXR) and ferredoxin (FDX). POR deficiency is a rare but well-described form of CAH characterized by impaired activity of 21-hydroxylase (P450c21, CYP21A2) and 17-hydroxylase/17,20-lyase (P450c17, CYP17A1); more severely affected individuals also have the Antley-Bixler skeletal malformation syndrome and disordered genital development in both sexes, and hence is easily recognized. The 17,20-lyase activity of P450c17 requires both POR and cytochrome b_5_ (b5), which promote electron transfer. Mutations of POR, b5, or P450c17 can cause selective 17,20-lyase deficiency. In addition to providing electrons to mitochondrial P450s, FDX, and FDXR are required for the synthesis of iron-sulfur clusters, which are used by many enzymes. Recent work has identified FDXR mutations in patients with visual impairment, optic atrophy, neuropathic hearing loss, and developmental delay, resembling the global neurologic disorders seen with mitochondrial diseases. Many of these patients have had life-threatening events or deadly infections, often without an apparent triggering event. Adrenal insufficiency has been predicted in such individuals but has only been documented recently. Neurologists, neonatologists, and geneticists should seek endocrine assistance in evaluating and treating patients with mutations in FDXR.

Steroidogenesis is the conversion of cholesterol into biologically active steroid hormones, (mineralocorticoids, glucocorticoids, androgens, estrogens, progestins), including conversion of a cholesterol precursor, 7-dehydrocholesterol, into biologically active 1,25-dihydroxyvitamin D. Most steroidogenic defects impair cortisol synthesis, with consequent pituitary hyperstimulation causing adrenal overgrowth, hence the term *congenital adrenal hyperplasia* (CAH), although not all defects in adrenal steroidogenesis cause adrenal overgrowth. Contemporary endocrinologists are attuned to the usual presenting features of 21-hydroxylase deficiency (21OHD, caused by mutations in *CYP21A2*), especially hyperandrogenism and salt loss. Human steroidogenesis is fairly well understood (1), but the clinical care of these disorders remains imperfect and evolving (2, 3) and important gaps in our understanding remain (4). New phenotypes have emerged beyond disorders of glucocorticoid and mineralocorticoid secretion and disorders of sexual development (DSD) associated with hyper- and hypo-androgenism. For example, among persons with salt-wasting 21OHD, about 10% will have CAH-X (5), which is 21OHD associated with the mild, “joint hypermobility” form of Ehlers-Danlos syndrome (EDS). This is because *CYP21A2* gene deletions frequently extend into the overlapping *TNX* gene encoding Tenascin-X, an extracellular matrix protein whose homozygous deficiency causes a severe form of EDS (6) and whose haploinsufficiency causes the mild, “joint hypermobility” form of EDS (7). Cytochrome b_5_ (b5) is familiar to endocrinologists for promoting the 17,20-lyase activity of P450c17 (CYP17A1), thus regulating the synthesis of androgen precursors (8, 9); but the principal action of b5 is the reduction of methemoglobin (10), and b5 deficiency causes DSD with methemoglobinemia (11, 12). Similarly, mutations in P450 oxidoreductase (POR) cause a form of CAH in which both 21-hydroxylase and 17-hydroxylase/17,20 lyase activities are impaired, but the diagnosis is usually first suspected because recessive POR deficiency also causes the Antley-Bixler skeletal malformation syndrome (13). POR serves to transfer electrons from reduced nicotinamide adenine dinucleotide (NADPH) to all cytochromes P450 located in endoplasmic reticulum (14, 15); the description of POR deficiency has drawn attention to the electron transfer proteins required by mitochondrial P450 enzymes: ferredoxin (FDX) and ferredoxin reductase (FDXR) (16). After years of searching, endocrine disorders associated with their mutations are emerging, further broadening the phenotypic spectrum of disorders of steroidogenesis. Here, we briefly review these factors and their disorders.

## Steroidogenesis

**Figure 1.**
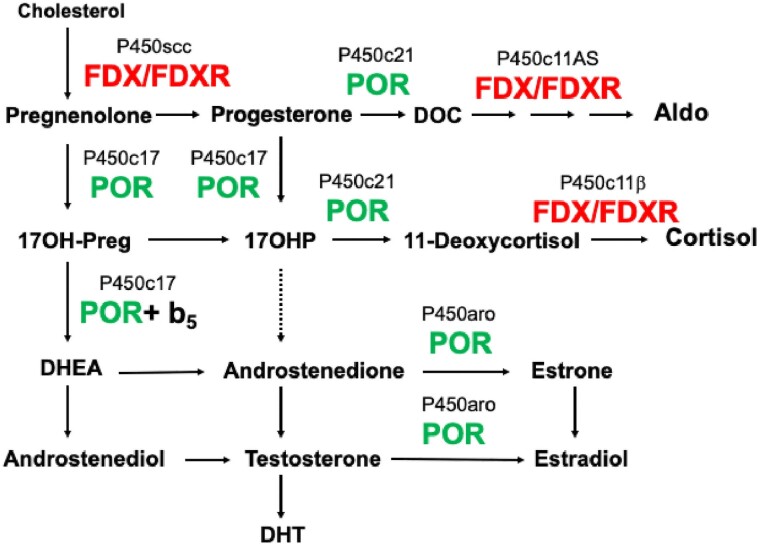
Simplified steroidogenic pathway. Only the human cytochrome P450 enzymes are shown; for figures showing all steroidogenic enzymes and other pathways, see (1). The “microsomal” (type 2) steroidogenic P450 enzymes are: P450c17 (17α-hydroxylase/17,20-lyase; CYP17A1), P450c21 (21-hydroxylase, CYP21A1), and P450aro (aromatase, CYP19A1); these 3 enzymes require electron donation from P450 oxidoreductase (POR). The 17,20-lyase activity of P450c17 is minimal in the absence of cytochrome *b5* (b_5_). The mitochondrial (type 1) steroidogenic P450 enzymes are: P450scc (cholesterol side-chain cleavage enzyme, CYP11A1), P450c11β (11β-hydroxylase, CYP11B1), and P450c11AS (aldosterone synthase, CYP11B2); these 3 enzymes require electron donation via ferredoxin (FDX) and ferredoxin reductase (FDXR). CYP11B2 catalyzes the 3 terminal steps (11-hydroxylation, 18-hydroxylation, and 18-methyl oxidase activity) in the production of aldosterone (Aldo) from deoxycorticosterone (DOC); each of these steps requires a pair of electrons donated from NADPH via FDX and FDXR.

The first enzymatic step in steroidogenesis is the conversion of cholesterol to pregnenolone in mitochondria, catalyzed by the cholesterol side-chain cleavage enzyme, P450scc (CYP11A1), encoded by the *CYP11A1* gene (Fig. 1). Expression of *CYP11A1* renders a cell “steroidogenic,” and the amount of P450scc produced determines a cell's steroidogenic capacity (17). In adrenal and gonadal steroidogenic cells that produce abundant steroids, mitochondrial cholesterol entry is facilitated by the steroidogenic acute regulatory protein (StAR) (18), which acts on the outer mitochondrial membrane to increase cholesterol import (19). In the placenta, brain, and skin, cholesterol enters mitochondria in steroidogenic cells without the action of StAR. This “StAR-independent steroidogenesis” is incompletely understood; it may involve mitochondrial entry of hydroxysterols, which are freely diffusible into mitochondria (18), or other proteins may substitute for StAR, such as placental MLN64 (20-22). Pregnenolone may be converted to progesterone by 3β-hydroxysteroid dehydrogenase, type 2 (3βHSD2), which is found in both the endoplasmic reticulum (ER) and mitochondria (23), where it appears to be located in the intermembranous space (24). Progesterone may be converted to glucocorticoids, mineralocorticoids, androgens, and estrogens by downstream enzymes, including cytoplasmic 17α-hydroxylase/17,20-lyase (P450c17, CYP17A1), 21-hydroxylase (P450c21, CYP21A2), and aromatase (P450aro, CYP19A1) and by mitochondrial 11β-hydroxylase (P450c11β, CYP11B1) and aldosterone synthase (P450c11AS, CYP11B2). The expression of these enzymes differs in different steroidogenic cell types, resulting in different steroidogenic pathways in different cell types (1).

## Cytochromes P450

**Figure 2.**
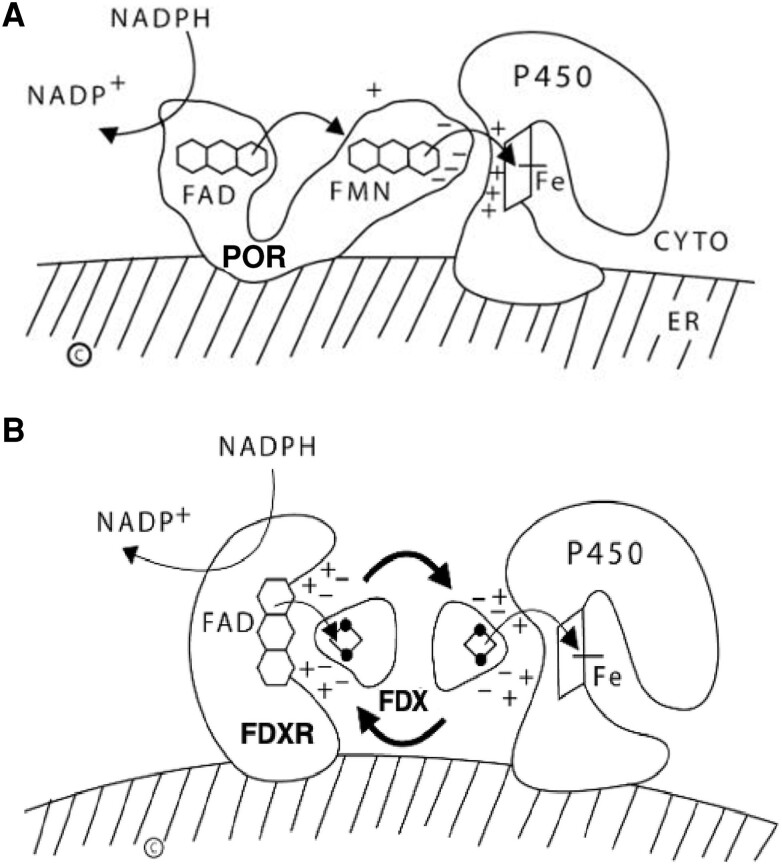
Diagrams of the cell biology of electron transfer to P450 enzymes. A, Microsomal (type 2) P450 enzymes. Both POR and the P450 are bound to the cytoplasmic aspect (CYTO) of the endoplasmic reticulum (ER). POR has 2 “wings,” one containing a flavin adenine dinucleotide (FAD) and one containing a flavin mononucleotide (FMN). NADPH interacts with and donates a pair of electrons to the FAD moiety. Electron receipt by the FAD elicits a conformational change, permitting the isoalloxazine rings of the FAD and FMN moieties to come sufficiently close together to permit the electrons to move from the FAD to the FMN. Electron receipt by the FMN permits the POR protein to revert to its original, open conformation; this permits the FMN domain to “dock” with the redox-partner binding site of the P450 by charge-charge interactions. The electrons reach the iron atom of the heme group of the P450, mediating catalysis. For some P450 reactions, notably the 17,20-lyase activity of human P450c17, cytochrome b_5_ promotes increased activity. B, Mitochondrial (type 1) P450 enzymes. Both ferredoxin reductase (FDXR) and the P450 are bound to the inner mitochondrial membrane, but ferredoxin (FDX) is not. NADPH donates a pair of electrons to the FAD moiety of FDXR; which then donates them to the 2Fe2S center of FDX (represented by the ball-and-stick glyph). The same surface of FDX interacts with both the FAD of FDXR and the redox-partner binding site of the P450 by electrostatic interactions. FDX thus acts as an indiscriminate electron-shuttling protein that can support the catalysis of any available type 1 P450. The electrons reach the heme iron of the P450 permitting catalysis.

There are 2 classes of human P450 enzymes: type 1 P450 enzymes in the mitochondria and type 2 P450 enzymes in the ER. The human genome has genes for 57 cytochrome P450s; 7 are type 1 and 50 are type 2 (25, 26). Among human steroidogenic enzymes, 5 are type 1 P450s: P450scc, P450c11β, P450c11AS, vitamin D 1α-hydroxylase (P450c1α, CYP27B1), and vitamin D 24-hydroxylase (P450c24, CYP24A1). Four other human steroidogenic enzymes are P450 type 2 P450s: P450c17, P450c21 (CYP21A2), P450aro (CYP19A1), and the principal vitamin D 25-hydroxylase (CYP2R1) (1); vitamin D 25-hydroxylation can also be catalyzed by CYP27A1 in mitochondria (27, 28) and CYP4A22 in the ER (29). Both types of P450 require that electrons from NADPH reach the heme iron of the P450, where catalysis occurs. To reach the P450 enzymes in the mitochondria or ER, electrons from NADPH must travel via different electron-transport chains (Fig. 2) (14).

## Electron Transfer to Microsomal P450s

All human type 2 P450 enzymes, including steroidogenic P450c17, P450c21, P450aro, and CYP2R1, as well as the other 46 microsomal P450s involved in drug/xenobiotic metabolism and synthesis of eicosanoids and leukotrienes, receive electrons from NADPH via P450 oxidoreductase (POR) (25). The activity of POR is not limited to P450s; POR also donates electrons to other enzymes, such as squalene monooxygenases, fatty acid elongase, heme oxygenase, b5, and to some small molecule substrates (15). POR has 2 distinct domains connected by a hinge; one domain contains a flavin adenine dinucleotide (FAD) moiety, the other contains a flavin mononucleotide (FMN) moiety (30). Before interacting with NADPH, POR is in an open conformation. When the electrons from NADPH are transferred to the FAD moiety, POR undergoes a conformational change that brings the FAD and FMN close together, permitting the electrons to travel to the FMN; this then returns POR to its open state. The FMN domain then interacts with the redox-partner binding site of the P450, permitting electron transfer to the heme iron in the P450, which then mediates catalysis (31) (Fig. 2A). Each P450 reaction cycle requires a pair of electrons, which are transferred from POR one at a time, but it remains unclear if both electrons are transferred by the same POR molecule or whether 2 separate interaction and electron transfer steps occur involving either the same or 2 different POR molecules (32).

## POR Deficiency

Because POR participates in so many essential biochemical processes, it was initially thought that POR deficiency would be lethal, and POR-knockout mice die during fetal development (33, 34). However, human POR deficiency “only” disrupts steroidogenesis and also causes a skeletal malformation syndrome (13, 35). The associated skeletal malformations, termed *Antley-Bixler syndrome* (ABS), apparently results from impaired activity of CYP26B1, a type 2 P450 that degrades retinoic acid (36). The bony derangements in ABS may include craniosynostosis, brachycephaly, radio-ulnar or radio-humeral synostosis, bowed femora, arachnodactyly, midface hypoplasia, proptosis, and choanal stenosis, with different patients manifesting somewhat different features (35). The ABS phenotype can be seen with either recessive mutations in POR or dominant, gain-of-function mutations in fibroblast growth factor receptor 2 (FGFR2) (35). Thus, recessive ABS with abnormal steroids and DSD in either sex is caused by mutations in POR, and dominant ABS without disordered steroidogenesis is caused by mutations in *FGFR2*. Defective signaling by *hedgehog* proteins secondary to POR-associated defects in cholesterol synthesis may also play a role. More than 200 mutations causing POR amino acid changes have been reported in over 250 patients, affecting various P450 enzymes to differing degrees, explaining the great clinical and hormonal variability in POR deficiency (37). More severe POR mutations cause the ABS phenotype, but mutations retaining ∼20% to 40% of activity may cause disturbed steroidogenesis without ABS (37), this may be considered “nonclassic POR deficiency.” The roles of these mutations in clinical disease, drug metabolism, and POR transcriptional regulation have been reviewed elsewhere (15, 16, 38) and will not be discussed here.

## Cytochrome b_5_

There are 2 human genes encoding cytochrome *b*_5_ (b5). *CYB5A* on chromosome 18q22.3, produces 2 alternatively spliced mRNAs. Exons 1 to 4 encode the 98-amino acid, soluble, cytosolic form found mainly in erythrocytes, where it reduces methemoglobin to hemoglobin. Exons 1 to 3 plus 5 and 6 encode the 134 amino acid form found in the ER; this is the predominant form in most cells, delivering electrons to microsomal desaturases that synthesize fatty acids, and is the overwhelmingly predominant and possibly only form of b5 involved in steroidogenesis (39-41). *CYB5B* on chromosome 16q22.1 encodes a 146-amino acid form found on the outer mitochondrial membrane and is widely expressed, including in steroidogenic tissues (41). Cytochrome b5 has a heme-binding domain and a structural core, from which the C-terminal membrane-anchoring helix extends. Cytochrome b5 can augment some P450 activities; apparently acting as an alternative donor of the second electron in the P450 cycle (42). However, some of the actions of b5 can be observed with apo-b5, which lacks a heme group and hence cannot transfer electrons (43). With human P450c17, b5 selectively stimulates 17,20-lyase activity but has negligible effects on 17-hydroxylase activity (8, 9).

Cytochrome b5 appears to enhance the interaction of P450c17 and POR, allosterically promoting more efficient electron transfer but not directly participating in electron transfer, as apo-b5 was as effective as holo-b5. This mechanism could account for b5 having no effect on the Km (Michaelis constant) of P450c17, while increasing the Vmax (maximum velocity) of the 17,20-lyase reaction (8, 9). Similarly, excess POR increases 17,20-lyase activity in the absence of b5 (44, 45), and mutations in the POR binding site of P450c17 selectively reduce 17,20-lyase activity (46, 47). Nevertheless, in recent work where the heme of b5 is replaced with a heme containing manganese rather than iron, this Mn-b5 was incapable of electron transfer and did not promote 17,20-lyase activity, even though the Mn-b5 interacted with the P450c17 (48, 49). Thus, current data suggest that b5 promotes 17,20-lyase activity by acting as an alternative electron donor. The 17,20-lyase activity of P450c17 can also be increased by phosphorylation of P450c17 on serine or threonine residues (50, 51), apparently catalyzed by p38α, a cAMP-dependent mitogen-activated protein kinase (52); the mechanism of this effect remains undetermined.

Because the reduction of methemoglobin is the principal physiologic role of b5, and methemoglobinemia is typically caused by deficiency of cytochrome b5 reductase, methemoglobinemia is a predictable consequence of b5 deficiency. The first report of b5 deficiency was in a patient with methemoglobinemia and DSD, but studies of steroidogenesis apparently were not done (10, 53). Since then, a small number of reports have described b5 deficiency in patients with apparent isolated 17,20-lyase deficiency, sometimes associated with methemoglobinemia (11, 12, 54). Most cases have been associated with 46,XY DSD, but normal development and fertility were reported with methemoglobinemia in a 24-year-old woman homozygous for b5 Y35X, which suggests possible compensation by *CYB5B* (55).

## Electron Transfer to Mitochondrial P450s

Mitochondrial, type 1 P450 enzymes receive electrons from NADPH via an electron transfer chain consisting of ferredoxin reductase (FDXR), which is loosely associated with the inner mitochondrial membrane, and ferredoxin (FDX) in the mitochondrial matrix (Fig. 2B) (1, 26). A pair of electrons from NADPH is accepted by FDXR (also termed *adrenodoxin reductase*), a 54-kDa flavoprotein encoded by the *FDXR* gene on chromosome 17q24 (56, 57). The flavin adenine dinucleotide (FAD) moiety of FDXR donates the electrons to the iron-sulfur (Fe-S) moiety of the 14 kDa FDX (also termed adrenodoxin). The same surface of FDX interacts sequentially with FDXR and with the recipient mitochondrial P450 (58). After FDX forms a 1:1 complex with FDXR it dissociates, then forms a 1:1 complex with the P450, thus acting as a diffusible electron shuttle (Fig. 2B). The human *FDXR* gene encodes 2 alternatively spliced mRNAs that can encode proteins of 491 or 497 amino acids (56, 57). Only the shorter protein is active in steroidogenesis (59); it is not known whether the longer form exerts an activity.

## Iron-Sulfur Proteins

Iron-sulfur clusters are prosthetic groups that are typically 2Fe-2S or 4Fe-4S, formed by reductive coupling of 2 2Fe-2S clusters (60), and are found in proteins that participate in electron transfer, such as FDX and several proteins in the mitochondrial respiratory chain complexes I, II, and III (61). The cellular processes producing such Fe-S clusters are highly conserved among eukaryotes, involving about 20 proteins that mediate 2 major steps: assembly of the Fe-S cluster on the scaffold protein ISCU (iron-sulfur cluster assembly enzyme), and transfer of the Fe-S cluster to a recipient protein (62-69). Reports of disorders of the assembly of Fe-S clusters and of the Fe-S proteins are increasingly reported in patients with neurologic and muscle diseases. The most widely studied protein participating in the assembly of Fe-S clusters is frataxin; expansion of a GAA repeat in intron 1 of its gene, *FRA*, causes Friedrich's ataxia (70). Mutations in ISCU affect assembly of Fe-S clusters in aconitase and succinate dehydrogenase, thus disrupting the Krebs cycle, primarily in skeletal muscle, causing exercise-induced lactic acidosis, muscle weakness, and, rarely, rhabdomyolysis (71-74).

## Ferredoxins (FDX1 and FDX2)

Mitochondrial ferredoxins carry electrons bound to a 2Fe-2S cluster. Ferredoxin was first identified in bacteria in the 1960s (75). In 1986, the amino acid sequences of bovine adrenal “adrenodoxin” and liver “hepatoredoxin” were found to be identical (76), suggesting there was only one mammalian ferredoxin, but that report, published in Russian, was not widely seen. Bovine adrenal “adrenodoxin” cDNA was cloned in 1985 (77), and human adrenal “adrenodoxin” (78) and placental “ferredoxin” (79) cDNAs were cloned in 1988; the 2 human sequences were identical, showing that the same gene was expressed in both tissues. Also in 1988, a single adrenodoxin gene was cloned (80) and located to chromosome 11q22 (81), which is predominantly, but not exclusively, expressed in steroidogenic tissues. That adrenodoxin/ferredoxin is now termed FDX1, encoded by the *FDX1* gene. The role of FDX1 in steroidogenesis suggests that *FDX1* mutations would be predicted to disrupt steroidogenesis similarly to *CYP11A1* (P450scc) mutations, but a human *FDX1* mutation has not (yet) been reported. Deletion of the related zebrafish *fdx1b* gene led to defective synthesis of cortisol and androgens (82, 83), but there are important differences in human and zebrafish steroidogenesis, hence the zebrafish results may not indicate the effects of a human *FDX1* mutation.

Yeast Yah1, the homolog of human FDX1, participates in synthesis of Fe-S clusters, but knockdown of FDX1 did not affect Fe-S cluster synthesis. Instead, the related *FDX2* gene (on chromosome 19p13.2), (formerly termed *FDX1L*), encodes FDX2, which supports Fe-S cluster synthesis but has little or no role in reducing mitochondrial P450s (84). The amino acid sequences of human FDX1 and FDX2 are 43% identical and 69% similar, and these FDX proteins have very similar three-dimensional structures (85), but their gene sequences are sufficiently different that *FDX2* was not detected in studies to determine the chromosomal location of *FDX1* (81). Both FDX1 and FDX2 participate in the synthesis of Fe-S clusters (65, 86), but FDX2 is more important for this activity, especially in the central nervous system, where *FDX2* is well-expressed and very little FDX1 is found. *FDX1* is abundantly expressed in steroidogenic tissues whereas *FDX2* is not, indicating that FDX1 is the principal form of ferredoxin involved in steroidogenesis (84). Nevertheless, it is unclear to what degree, if any, FDX1 and FDX2 might be able to substitute for one another in clinical situations where one is deficient.

*FDX2* has been implicated in several neurological diseases (87, 88), including Friedreich's ataxia and Parkinson's disease (89). Specific role(s) for FDX2 in these disorders remain uncertain, but 2 studies reported mitochondrial muscle myopathy with or without optic atrophy and reversible leukoencephalopathy (MEOAL; OMIM #251900) in patients with *FDX2* mutations. A 15-year-old girl born to consanguineous parents had normal psychomotor development to age 12, then had episodes of proximal muscle weakness, myoglobinuria, lactic acidosis, and increased serum creatine kinase; a homozygous missense mutation was identified by whole exome sequencing in the initiation codon of the *FDX1L* (*FDX2*) gene (90). FDX2 protein was essentially undetectable in a muscle biopsy or cultured fibroblasts, and activities of aconitase and respiratory complexes I, II, and III, all of which have Fe-S clusters, were impaired. Six similar patients from 2 families presented variably from early childhood to adulthood with nonprogressive optic atrophy, muscle weakness, cramps, and myalgia, often associated with exercise, infection, or low temperature; other studies, including muscle biopsies, implicated disordered mitochondrial function (91). Patient DNA was homozygous for *FDX2* missense mutations, and RNA and protein blotting studies suggested the mutant FDX2 protein was unstable. Thus, mutations in *FDX2* appear to cause neurologic impairments, apparently related to impaired synthesis of Fe-S clusters, yielding global mitochondrial dysfunction. Studies of steroidogenesis were not done in these patients.

## Ferredoxin Reductase

In addition to FDX1, FDX2, and other proteins, the synthesis of Fe-S clusters requires FDXR (64, 67-69). Early studies showed low levels of ferredoxin reductase (FDXR) mRNA in all tissues, although expression in adrenal and testis was about 100-fold greater (92). Because both FDX1 and FDX2 play a role in the biogenesis of Fe-S centers and there is only one *FDXR* gene (57), one might predict that *FDXR* mutations would also affect Fe-S synthesis and result in a phenotype similar to that of FDX2 deficiency (ie, MEOAL), but also with impaired steroidogenesis. Knockdown of the genes for FDX1, FDX2, or FDXR in human cell lines reduced Fe-S cluster synthesis and impaired several enzymes that rely on Fe-S clusters for activity, and also depleted cytosolic iron, causing mitochondrial iron overload (65, 86). Thus, interference with *FDX1*, *FDX2*, or *FDXR*, disrupts the synthesis and/or assembly of Fe-S clusters and disrupts intracellular iron homeostasis.

## Animal Models of FDXR Deficiency

Long before human *FDXR* mutations were sought, the first report of a genetic defect in FDXR was the identification of the *dare* mutation and gene in *Drosophila melanogaster* (91). The *dare* mutant was identified in a screen of mutations that affected olfactory-driven learning and memory; this *Drosophila* mutant phenotype was named “*dare*” from defective in avoidance of repellants. When the responsible gene was cloned and sequenced, it was found to be *Drosophila* FDXR; as FDXR was then generally termed *adrenodoxin reductase*, the name “*dare*” was repurposed as *Drosophila*  adrenodoxin reductase (93). Insect development and metamorphosis, including sexual development and cuticle (exoskeleton) development, depend on the steroid hormone ecdysone; the developmental arrest characteristic of the *dare* phenotype could be rescued by feeding mutant larvae 20-hydroxyecdysone. In situ hybridization showed that *dare* expression was confined to steroidogenic tissues: the parathoracic cells of the larval “ring gland” and the ovary, where FDXR is required for egg chamber development (94). In adult flies, timed *dare* mutations caused degeneration of the adult nervous system. This *dare* was the first gene/factor discovered in the insect pathway from cholesterol to ecdysone, the so-called “Halloween pathway” now known to include both microsomal and mitochondrial P450 enzymes, as well as short-chain dehydrogenases—much like human steroidogenic pathways, but without cleavage of the side chain of cholesterol (95-97).

Mice are the most widely used mammalian model for human biology and disease. Fairfield and 38 collaborators used ethyl-nitrosourea mutagenesis to create 172 mouse (C57BL/6J) mutants and performed exome sequencing to identify mutations in 91 of the strains produced; one strain was homozygous for the *Fdxr* mutation R389Q (98). The principal phenotype in these animals was described as “behavioral; neurological.” These mice have decreased visual acuity, a progressive gait disorder, and Fdxr activity that is reduced to between ∼33% and 50% of normal, varying with the tissue assessed (99). Mouse Fdxr R389Q corresponds to human R392Q, now known to be a cause of FDXR deficiency (99).

## Human FDXR Deficiency

From 2017 to 2023, at least 77 patients from more than 50 families were described carrying 59 different biallelic (homozygous or compound heterozygous) *FDXR* mutations causing a generalized mitochondriopathy, variably presenting with optic atrophy, retinal dystrophy, neuropathic hearing loss, developmental delay, mild movement disorders, and even Leigh syndrome with infantile-onset encephalopathy and death (99-110); this disorder has been termed *FDXR-related mitochondriopathy* (FRM) (110). Most families with FRM were not known to be related to one another, but the frequent identification of specific mutations in some studies suggested there were local founder effects or unknown consanguinity. Among 13 families (26 alleles) in a study published in 2017, 11 alleles carried the missense mutation R392W (99), and among 8 families (16 alleles) in a study published in 2021, 5 alleles carried P372H (104); however, neither of these mutations was found in any other report, indicating that they are not mutational “hot spots.” Another study screened 2186 patients with hereditary optic neuropathy: 1126 had apparent mitochondrial inheritance, with mitochondrial defects found in 199, and 1680 had apparent autosomal disease, with mutations found in 451. Among those with autosomal disease, mutations in only 10 genes accounted for 434/451 (96%) of those patients, and 5/434 of these patients had mutations in FDXR; all of whom also had deafness, and most were < 10 years old (109). In a recent study, FDXR R386W was found in one or both alleles in ∼25% of affected individuals; most were Hispanic, many of Mexican heritage. The genome databases MCPS and gnomAD reported allele frequencies of 0.0027 and 0.001274, respectively for Indigenous Mexican and Latino/Admixed American population, indicating carrier frequencies of 1/185 and 1/394 in the Indigenous and Admixed Mexican populations, respectively (110), thus FDXR deficiency may be more common in this population. All patients who have been reported on to date had at least one allele that retained partial activity; the same situation is found among patients with POR deficiency: no patient has been reported with wholly null mutations on both alleles. These observations suggest that homozygosity or compound heterozygosity for null alleles for either POR or FDXR may be lethal in embryonic or fetal life.

Cell culture studies are consistent with the observations from human genetics. Knockdown of FDXR in HeLa cells and in human K562 erythroid cells leads to iron overload (86); similarly, primary cultures of fibroblasts from patients with FRM had reduced FDXR activity and increased production of reactive oxygen species (99). Thus clinical, genetic, biochemical, and cell biologic data show that FDXR deficiency causes a generalized mitochondrial disorder, FRM, primarily seen in the central nervous system, that shares many features with FDX2 deficiency and other mitochondrial disorders. However, from a clinical endocrine perspective, the most remarkable feature of these reports was the absence of studies assessing clinical adrenal function or addressing the obligatory role of FDXR in steroid hormone synthesis; adrenal insufficiency had been predicted in these patients (4, 16).

A very recent study has now shown such adrenal insufficiency in 2 severely affected siblings with FRM, DSD, and hypertension, with a steroid pattern suggesting 11-hydroxylase deficiency (111). This study also showed that the previously reported mice carrying the *Fdxr* R389Q mutation also had impaired adrenal function, with impaired corticosterone synthesis, indicative of defective FDXR-supported activity of 11β-hydroxylase (CYP11B1). Review of the previously published cases of FRM showed that 20/77 (26%) of these patients also had a history of severe, often life-threatening events or deadly infections suggestive of adrenal insufficiency (111). Only 3 steroidogenic P450 enzymes, the cholesterol side-chain cleavage enzyme, P450scc (CYP11A1), 11β-hydroxylase, P450c11β (CYP11B1), and aldosterone synthase, P450c11AS (CYP11B2), reside in mitochondria and require electron donation from FDX and FDXR (Fig. 1). P450scc is the rate-limiting enzyme in steroidogenesis (1), hence interference with its activity by FDXR deficiency must impair all steroidogenesis, but as 100-fold more cortisol is produced than aldosterone (1), glucocorticoid deficiency is the predictable result. However, cortisol synthesis also requires P450c11β, and indeed the 2 index FDXR-deficient patients had clear signs of 11β-hydroxylase deficiency (mineralocorticoid hypertension secondary to overproduction of deoxycorticosterone [DOC], and increased plasma 11-deoxycortisol) (111). FDXR is also required by P450c11AS, which catalyzes the last 3 steps in the production of aldosterone from DOC (1) (Fig. 1); impairment of P450c11AS might be expected to manifest with salt loss, but overproduction of DOC from impairment of P450c11β would mask aldosterone deficiency. The 2 FDXR-deficient infants did not survive long enough to permit investigation of their renin-angiotensin-aldosterone axis (111).

Thus, neonatologists, neurologists, and geneticists caring for these patients must become familiar with the life-threatening possibility of adrenal insufficiency when FRM is suspected, and seek endocrine assistance to evaluate this possibility, as treating adrenal insufficiency may prolong survival and quality of life. Future studies should include clinical investigation of adrenal reserve by performing adrenocorticotropic hormone (ACTH) stimulation tests with, at minimum, measurement of cortisol and 11-deoxycortisol; such ACTH testing should now be routine when a disorder of FDX or FDXR is suspected. Cell biologic studies of steroidogenesis and FDXR function are essential, but the logical approach of transfecting nonsteroidogenic cells with vectors expressing a mitochondrial P450 plus either wild-type or mutant FDXR was unreliable because of high background FDXR activity in cultured cells. Instead, induced pluripotent cells derived from FDXR-deficient patient fibroblasts that had been differentiated in vitro toward an adrenal-like lineage were needed to show the effects of FDXR mutations on steroidogenesis (111). We speculate that patients with less severe *FDXR* mutations that retain partial activity will have compensated adrenal insufficiency, as seen in the nonclassic forms of 21-hydroxylase deficiency (112), lipoid CAH (113), P450scc deficiency (114), and POR deficiency without ABS (13, 35, 38, 115). However, while the adrenal insufficiency of those nonclassic forms of CAH appears to be stable as the patients age, we speculate that the clinical findings in “nonclassic FDXR deficiency,” like many other mitochondriopathies, will worsen with age, requiring long-term monitoring of adrenal function.

## Conclusions

Disorders of the factors that transfer electrons from NADPH to steroidogenic cytochrome P450 enzymes are a newly recognized group of disorders of steroidogenesis, presenting similarly to CAH and adrenal insufficiency. Mutations in P450 oxidoreductase were first described in 2004, and are now well-characterized clinically, genetically, and biochemically. Mutations in cytochrome b5 were first described in 2010, causing isolated 17,20-lyase deficiency, but this remains one of the rarest disorders in steroidogenesis. Mutations in FDXR were first reported in 2017, causing visual impairment, neuropathic hearing loss, and other features of mitochondriopathy, but the predictable steroidogenic consequences were not sought. Recent work has now reported adrenal insufficiency in patients with severe FDXR deficiency; additional careful clinical studies of adrenal and gonadal steroidogenesis in these patients is needed, and physicians caring for these patients must become aware of the possibility of potentially lethal adrenal insufficiency in these patients. Mutations of FDX2 (which does not appear to participate in steroidogenesis) are associated with neurologic disorders but not with adrenal insufficiency. Mutations in FDX1, which *does* participate in steroidogenesis have not (yet) been reported, but we predict that they will be found and will cause adrenal insufficiency. Finally, most genetic disorders—not just those affecting steroidogenesis—are first identified in more severely affected individuals, with milder cases and “nonclassical” disease generally being reported later; thus the severely affected FDXR-deficient patients initially reported (111) probably do not represent a “typical picture” of FDXR deficiency; astute physicians should be alert for milder forms of this disorder.

## Disclosures

The authors report no conflict of interest.

## Data Availability

Data sharing is not applicable to this article as no datasets were generated or analyzed during the current study.

## Abbreviations

21OHD: 21-hydroxylase deficiency
ABS: Antley-Bixler syndrome
b5: cytochrome *b_5_*
CAH: congenital adrenal hyperplasia
DOC: deoxycorticosterone
DSD: disorder/difference of sexual development
EDS: Ehlers-Danlos syndrome
ER: endoplasmic reticulum
FAD: flavin adenine dinucleotide
FDX: ferredoxin
FDXR: ferredoxin reductase
FGFR2: fibroblast growth factor receptor 2
FMN: flavin mononucleotide
FRM: FDXR-related mitochondriopathy
ISCU: iron-sulfur cluster assembly enzyme
MEOAL: mitochondrial muscle myopathy with or without optic atrophy and reversible leukoencephalopathy
NADPH: reduced nicotinamide dinucleotide phosphate
POR: P450 oxidoreductase
StAR: steroidogenic acute regulatory protein

## References

[1] Miller WL, Auchus RJ. The molecular biology, biochemistry, and physiology of human steroidogenesis and its disorders. Endocr Rev. 2011;32(1):81‐151. 21051590 10.1210/er.2010-0013 PMC3365799

[2] Speiser PW, Arlt W, Auchus RJ, et al Congenital adrenal hyperplasia due to steroid 21-hydroxylase deficiency: an endocrine society clinical practice guideline. J Clin Endocrinol Metab. 2018;103(11):4043‐4088. 30272171 10.1210/jc.2018-01865 PMC6456929

[3] Claahsen-van der Grinten HL, Speiser PW, Ahmed SF, et al Congenital adrenal hyperplasia - current insights in pathophysiology, diagnostics and management. Endocr Rev. 2021;43(1):91‐159. 10.1210/endrev/bnab016 PMC8755999 33961029

[4] Miller WL . Steroidogenesis: unanswered questions. Trends Endocrinol Metab. 2017;28(11):771‐793. 29031608 10.1016/j.tem.2017.09.002

[5] Miller WL, Merke DP. Tenascin-X, congenital adrenal hyperplasia, and the CAH-X syndrome. Horm Res Paediatr. 2018;89(5):352‐361. 29734195 10.1159/000481911 PMC6057477

[6] Schalkwijk J, Zweers MC, Steijlen PM, et al A recessive form of the Ehlers-Danlos syndrome caused by tenascin-X deficiency. N Engl J Med. 2001;345(16):1167‐1175. 11642233 10.1056/NEJMoa002939

[7] Zweers MC, Bristow J, Steijlen PM, et al Haploinsufficiency of TNXB is associated with hypermobility type of Ehlers-Danlos syndrome. Am J Hum Genet. 2003;73(1):214‐217. 12865992 10.1086/376564 PMC1180584

[8] Auchus RJ, Lee TC, Miller WL. Cytochrome b5 augments the 17,20-lyase activity of human P450c17 without direct electron transfer. J Biol Chem. 1998;273(6):3158‐3165. 9452426 10.1074/jbc.273.6.3158

[9] Pandey AV, Miller WL. Regulation of 17,20 lyase activity by cytochrome b5 and by serine phosphorylation of P450c17. J Biol Chem. 2005;280(14):13265‐13271. 15687493 10.1074/jbc.M414673200

[10] Hegesh E, Hegesh J, Kaftory A. Congenital methemoglobinemia with a deficiency of cytochrome b5. N Engl J Med. 1986;314(12):757‐761. 3951505 10.1056/NEJM198603203141206

[11] Kok RC, Timmerman MA, Wolffenbuttel KP, Drop SL, de Jong FH. Isolated 17,20-lyase deficiency due to the cytochrome b5 mutation W27X. J Clin Endocrinol Metab. 2010;95(3):994‐999. 20080843 10.1210/jc.2008-1745

[12] Idkowiak J, Randell T, Dhir V, et al A missense mutation in the human cytochrome b5 gene causes 46,XY disorder of sex development due to true isolated 17,20 lyase deficiency. J Clin Endocrinol Metab. 2012;97(3):E465‐E475. 22170710 10.1210/jc.2011-2413 PMC3388247

[13] Fluck CE, Tajima T, Pandey AV, et al Mutant P450 oxidoreductase causes disordered steroidogenesis with and without Antley-Bixler syndrome. Nat Genet. 2004;36(3):228‐230. 14758361 10.1038/ng1300

[14] Miller WL . Regulation of steroidogenesis by electron transfer. Endocrinology. 2005;146(6):2544‐2550. 15774560 10.1210/en.2005-0096

[15] Pandey AV, Fluck CE. NADPH P450 oxidoreductase: structure, function, and pathology of diseases. Pharmacol Ther. 2013;138(2):229‐254. 23353702 10.1016/j.pharmthera.2013.01.010

[16] Miller WL . Steroidogenic electron-transfer factors and their diseases. Ann Pediatr Endocrinol Metab. 2021;26(3):138‐148. 34610701 10.6065/apem.2142154.077 PMC8505039

[17] Voutilainen R, Tapanainen J, Chung BC, Matteson KJ, Miller WL. Hormonal regulation of P450scc (20,22-desmolase) and P450c17 (17α-hydroxylase/17,20-lyase) in cultured human granulosa cells. J Clin Endocrinol Metab. 1986;63(1):202‐207. 3011839 10.1210/jcem-63-1-202

[18] Miller WL, Bose HS. Early steps in steroidogenesis: intracellular cholesterol trafficking. J Lipid Res. 2011;52(12):2111‐2135. 21976778 10.1194/jlr.R016675 PMC3283258

[19] Bose HS, Lingappa VR, Miller WL. Rapid regulation of steroidogenesis by mitochondrial protein import. Nature. 2002;417(6884):87‐91. 11986670 10.1038/417087a

[20] Watari H, Arakane F, Moog-Lutz C, et al MLN64 contains a domain with homology to the steroidogenic acute regulatory protein (StAR) that stimulates steroidogenesis. Proc Natl Acad Sci U S A. 1997;94(16):8462‐8467. 9237999 10.1073/pnas.94.16.8462 PMC22957

[21] Bose HS, Whittal RM, Huang MC, Baldwin MA, Miller WL. N-218 MLN64, a protein with StAR-like steroidogenic activity, is folded and cleaved similarly to StAR. Biochemistry. 2000;39(38):11722‐11731. 10995240 10.1021/bi000911l

[22] Tuckey RC, Bose HS, Czerwionka I, Miller WL. Molten globule structure and steroidogenic activity of N-218 MLN64 in human placental mitochondria. Endocrinology. 2004;145(4):1700‐1707. 14715710 10.1210/en.2003-1034

[23] Cherradi N, Rossier MF, Vallotton MB, et al Submitochondrial distribution of three key steroidogenic proteins (steroidogenic acute regulatory protein and cytochrome P450scc and 3(-hydroxysteroid dehydrogenase isomerase enzymes) upon stimulation by intracellular calcium in adrenal glomerulosa cells. J Biol Chem. 1997;272(12):7899‐7907. 9065457 10.1074/jbc.272.12.7899

[24] Thomas JL, Bose HS. Regulation of human 3β-hydroxysteroid dehydrogenase type-2 (3βHSD2) by molecular chaperones and the mitochondrial environment affects steroidogenesis. J Steroid Biochem Mol Biol. 2015;151:74‐84. 25448736 10.1016/j.jsbmb.2014.11.018

[25] Nebert DW, Wikvall K, Miller WL. Human cytochromes P450 in health and disease. Philos Trans R Soc Lond B Biol Sci. 2013;368(1612):20120431. 23297354 10.1098/rstb.2012.0431 PMC3538421

[26] Omura T . Mitochondrial P450s. Chem Biol Interact. 2006;163(1–2):86‐93. 16884708 10.1016/j.cbi.2006.06.008

[27] Usui E, Noshiro M, Okuda K. Molecular cloning of cDNA for vitamin D3 25-hydroxylase from rat liver mitochondria. FEBS Lett. 1990;262(1):135‐138. 2318307 10.1016/0014-5793(90)80172-f

[28] Su P, Rennert H, Shayiq RM, et al A cDNA encoding a rat mitochondrial cytochrome P450 catalyzing both the 26-hydroxylation of cholesterol and 25-hydroxylation of vitamin D3: gonadotropic regulation of the cognate mRNA in ovaries. DNA Cell Biol. 1990;9(9):657‐667. 2175615 10.1089/dna.1990.9.657

[29] Duan X, Zhang Y, Xu T. CYP4A22 loss-of-function causes a new type of vitamin D-dependent rickets (VDDR1C). J Bone Miner Res. 2024;39(7):967‐979. 38847469 10.1093/jbmr/zjae084

[30] Wang M, Roberts DL, Paschke R, Shea TM, Masters BS, Kim JJ. Three-dimensional structure of NADPH-cytochrome P450 reductase: prototype for FMN- and FAD-containing enzymes. Proc Natl Acad Sci U S A. 1997;94(16):8411‐8416. 9237990 10.1073/pnas.94.16.8411 PMC22938

[31] Ellis J, Gutierrez A, Barsukov IL, Huang WC, Grossmann JG, Roberts GC. Domain motion in cytochrome P450 reductase: conformational equilibria revealed by NMR and small-angle x-ray scattering. J Biol Chem. 2009;284(52):36628‐36637. 19858215 10.1074/jbc.M109.054304 PMC2794777

[32] Guengerich FP . Mechanisms of cytochrome P450-catalyzed oxidations. ACS Catal. 2018;8(12):10964‐10976. 31105987 10.1021/acscatal.8b03401 PMC6519473

[33] Shen AL, O’Leary KA, Kasper CB. Association of multiple developmental defects and embryonic lethality with loss of microsomal NADPH-cytochrome P450 oxidoreductase. J Biol Chem. 2002;277(8):6536‐6541. 11742006 10.1074/jbc.M111408200

[34] Otto DM, Henderson CJ, Carrie D, et al Identification of novel roles of the cytochrome P450 system in early embryogenesis: effects on vasculogenesis and retinoic acid homeostasis. Mol Cell Biol. 2003;23(17):6103‐6116. 12917333 10.1128/MCB.23.17.6103-6116.2003 PMC180925

[35] Huang N, Pandey AV, Agrawal V, et al Diversity and function of mutations in P450 oxidoreductase in patients with antley-bixler syndrome and disordered steroidogenesis. Am J Hum Genet. 2005;76(5):729‐749. 15793702 10.1086/429417 PMC1199364

[36] Laue K, Pogoda HM, Daniel PB, et al Craniosynostosis and multiple skeletal anomalies in humans and zebrafish result from a defect in the localized degradation of retinoic acid. Am J Hum Genet. 2011;89(5):595‐606. 22019272 10.1016/j.ajhg.2011.09.015 PMC3213388

[37] Flück CE, Rojas Velazquez MN, Pandey AV. Chapter 12 - P450 oxidoreductase deficiency. In: New MI, editor. Genetic Steroid Disorders. 2nd ed. Academic Press; 2023: 239‐264.

[38] Miller WL, Agrawal V, Sandee D, et al Consequences of POR mutations and polymorphisms. Mol Cell Endocrinol. 2011;336(1–2):174‐179. 21070833 10.1016/j.mce.2010.10.022 PMC4632974

[39] Giordano SJ, Steggles AW. The human liver and reticulocyte cytochrome b5 mRNAs are products from a single gene. Biochem Biophys Res Commun. 1991;178(1):38‐44. 1712589 10.1016/0006-291x(91)91776-9

[40] Schenkman JB, Jansson I. The many roles of cytochrome b5. Pharmacol Ther. 2003;97(2):139‐152. 12559387 10.1016/s0163-7258(02)00327-3

[41] Storbeck KH, Swart AC, Fox CL, Swart P. Cytochrome b5 modulates multiple reactions in steroidogenesis by diverse mechanisms. J Steroid Biochem Mol Biol. 2015;151:66‐73. 25446886 10.1016/j.jsbmb.2014.11.024

[42] Bridges A, Gruenke L, Chang YT, Vakser IA, Loew G, Waskell L. Identification of the binding site on cytochrome P450 2B4 for cytochrome b5 and cytochrome P450 reductase. J Biol Chem. 1998;273(27):17036‐17049. 9642268 10.1074/jbc.273.27.17036

[43] Yamazaki H, Johnson WW, Ueng YF, Shimada T, Guengerich FP. Lack of electron transfer from cytochrome b(5) in stimulation of catalytic activities of cytochrome P450 3A4 - characterization of a reconstituted cytochrome P450 3A4 NADPH-cytochrome P450 reductase system and studies with apo-cytochrome b(5). J Biol Chem. 1996;271(44):27438‐27444. 8910324 10.1074/jbc.271.44.27438

[44] Yanagibashi K, Hall PF. Role of electron transport in the regulation of the lyase activity of C21 side-chain cleavage P-450 from porcine adrenal and testicular microsomes. J Biol Chem. 1986;261(18):8429‐8433. 3487544

[45] Lin D, Black SM, Nagahama Y, Miller WL. Steroid 17α-hydroxylase and 17,20-lyase activities of P450c17: contributions of serine106 and P450 reductase. Endocrinology. 1993;132(6):2498‐2506. 8504753 10.1210/endo.132.6.8504753

[46] Geller DH, Auchus RJ, Mendonca BB, Miller WL. The genetic and functional basis of isolated 17,20-lyase deficiency. Nat Genet. 1997;17(2):201‐205. 9326943 10.1038/ng1097-201

[47] Geller DH, Auchus RJ, Miller WL. P450c17 mutations R347H and R358Q selectively disrupt 17,20-lyase activity by disrupting interactions with P450 oxidoreductase and cytochrome b5. Mol Endocrinol. 1999;13(1):167‐175. 9892022 10.1210/mend.13.1.0219

[48] Duggal R, Liu Y, Gregory MC, Denisov IG, Kincaid JR, Sligar SG. Evidence that cytochrome b5 acts as a redox donor in CYP17A1 mediated androgen synthesis. Biochem Biophys Res Commun. 2016;477(2):202‐208. 27297105 10.1016/j.bbrc.2016.06.043 PMC4935565

[49] Duggal R, Denisov IG, Sligar SG. Cytochrome b(5) enhances androgen synthesis by rapidly reducing the CYP17A1 oxy-complex in the lyase step. FEBS Lett. 2018;592(13):2282‐2288. 29888793 10.1002/1873-3468.13153 PMC6369587

[50] Zhang LH, Rodriguez H, Ohno S, Miller WL. Serine phosphorylation of human P450c17 increases 17,20-lyase activity: implications for adrenarche and the polycystic ovary syndrome. Proc Natl Acad Sci U S A. 1995;92(23):10619‐10623. 7479852 10.1073/pnas.92.23.10619 PMC40663

[51] Pandey AV, Mellon SH, Miller WL. Protein phosphatase 2A and phosphoprotein SET regulate androgen production by P450c17. J Biol Chem. 2003;278(5):2837‐2844. 12444089 10.1074/jbc.M209527200

[52] Tee MK, Miller WL. Phosphorylation of human cytochrome P450c17 by p38( selectively increases 17,20 lyase activity and androgen biosynthesis. J Biol Chem. 2013;288(33):23903‐23913. 23836902 10.1074/jbc.M113.460048 PMC3745337

[53] Giordano SJ, Kaftory A, Steggles AW. A splicing mutation in the cytochrome b5 gene from a patient with congenital methemoglobinemia and pseudohermaphrodism. Hum Genet. 1994;93(5):568‐570. 8168836 10.1007/BF00202825

[54] Shaunak M, Taylor NF, Hunt D, Davies JH. Isolated 17, 20–lyase deficiency secondary to a novel CYB5A variant: comparison of steroid metabolomic findings with published cases provides diagnostic guidelines and greater insight into its biological role. Horm Res Paediatr. 2020;93(7–8):483‐496. 33626548 10.1159/000512372

[55] Leung MT, Cheung HN, Iu YP, Choi CH, Tiu SC, Shek CC. Isolated 17,20-lyase deficiency in a CYB5A mutated female with normal sexual development and fertility. J Endocr Soc. 2020;4(2):bvz016. 32051920 10.1210/jendso/bvz016 PMC7007803

[56] Solish SB, Picado-Leonard J, Morel Y, et al Human adrenodoxin reductase: two mRNAs encoded by a single gene on chromosome 17cen----q25 are expressed in steroidogenic tissues. Proc Natl Acad Sci U S A. 1988;85(19):7104‐7108. 2845396 10.1073/pnas.85.19.7104 PMC282132

[57] Lin D, Shi YF, Miller WL. Cloning and sequence of the human adrenodoxin reductase gene. Proc Natl Acad Sci U S A. 1990;87(21):8516‐8520. 2236061 10.1073/pnas.87.21.8516 PMC54987

[58] Coghlan VM, Vickery LE. Site-specific mutations in human ferredoxin that affect binding to ferredoxin reductase and cytochrome P450scc. J Biol Chem. 1991;266(28):18606‐18612. 1917982

[59] Brandt ME, Vickery LE. Expression and characterization of human mitochondrial ferredoxin reductase in Escherichia coli. Arch Biochem Biophys. 1992;294(2):735‐740. 1567230 10.1016/0003-9861(92)90749-m

[60] Chandramouli K, Unciuleac MC, Naik S, Dean DR, Huynh BH, Johnson MK. Formation and properties of [4Fe-4S] clusters on the IscU scaffold protein. Biochemistry. 2007;46(23):6804‐6811. 17506525 10.1021/bi6026659

[61] Bak DW, Elliott SJ. Alternative FeS cluster ligands: tuning redox potentials and chemistry. Curr Opin Chem Biol. 2014;19:50‐58. 24463764 10.1016/j.cbpa.2013.12.015

[62] Li K, Tong WH, Hughes RM, Rouault TA. Roles of the mammalian cytosolic cysteine desulfurase, ISCS, and scaffold protein, ISCU, in iron-sulfur cluster assembly. J Biol Chem. 2006;281(18):12344‐12351. 16527810 10.1074/jbc.M600582200

[63] Rouault TA . Biogenesis of iron-sulfur clusters in mammalian cells: new insights and relevance to human disease. Dis Model Mech. 2012;5(2):155‐164. 22382365 10.1242/dmm.009019 PMC3291637

[64] Braymer JJ, Lill R. Iron-sulfur cluster biogenesis and trafficking in mitochondria. J Biol Chem. 2017;292(31):12754‐12763. 28615445 10.1074/jbc.R117.787101 PMC5546016

[65] Cai K, Tonelli M, Frederick RO, Markley JL. Human mitochondrial ferredoxin 1 (FDX1) and ferredoxin 2 (FDX2) both bind cysteine desulfurase and donate electrons for iron-sulfur cluster biosynthesis. Biochemistry. 2017;56(3):487‐499. 28001042 10.1021/acs.biochem.6b00447 PMC5267338

[66] Vanlander AV, Van Coster R. Clinical and genetic aspects of defects in the mitochondrial iron-sulfur cluster synthesis pathway. J Biol Inorg Chem. 2018;23(4):495‐506. 29623423 10.1007/s00775-018-1550-z PMC6006192

[67] Maio N, Rouault TA. Outlining the complex pathway of mammalian Fe-S cluster biogenesis. Trends Biochem Sci. 2020;45(5):411‐426. 32311335 10.1016/j.tibs.2020.02.001 PMC8349188

[68] Maio N, Jain A, Rouault TA. Mammalian iron-sulfur cluster biogenesis: recent insights into the roles of frataxin, acyl carrier protein and ATPase-mediated transfer to recipient proteins. Curr Opin Chem Biol. 2020;55:34‐44. 31918395 10.1016/j.cbpa.2019.11.014 PMC7237328

[69] Zhang W, Xu L, Zhao H, Li K. Mammalian mitochondrial iron-sulfur cluster biogenesis and transfer and related human diseases. Biophys Rep. 2021;7(2):127‐141. 37288145 10.52601/bpr.2021.200038 PMC10235907

[70] Campuzano V, Montermini L, Molto MD, et al Friedreich's ataxia: autosomal recessive disease caused by an intronic GAA triplet repeat expansion. Science. 1996;271(5254):1423‐1427. 8596916 10.1126/science.271.5254.1423

[71] Mochel F, Knight MA, Tong WH, et al Splice mutation in the iron-sulfur cluster scaffold protein ISCU causes myopathy with exercise intolerance. Am J Hum Genet. 2008;82(3):652‐660. 18304497 10.1016/j.ajhg.2007.12.012 PMC2427212

[72] Olsson A, Lind L, Thornell LE, Holmberg M. Myopathy with lactic acidosis is linked to chromosome 12q23.3–24.11 and caused by an intron mutation in the ISCU gene resulting in a splicing defect. Hum Mol Genet. 2008;17(11):1666‐1672. 18296749 10.1093/hmg/ddn057

[73] Kollberg G, Tulinius M, Melberg A, et al Clinical manifestation and a new ISCU mutation in iron-sulphur cluster deficiency myopathy. Brain. 2009;132(Pt 8):2170‐2179. 19567699 10.1093/brain/awp152

[74] Legati A, Reyes A, Ceccatelli Berti C, et al A novel de novo dominant mutation in ISCU associated with mitochondrial myopathy. J Med Genet. 2017;54(12):815‐824. 29079705 10.1136/jmedgenet-2017-104822 PMC5740555

[75] Valentine RC . Bacterial ferredoxin. Bacteriol Rev. 1964;28(4):497‐517. 14244728 10.1128/br.28.4.497-517.1964 PMC441251

[76] Chashchin VL, Lapko VN, Adamovich TB, Kirillova NM, Lapko AG, Akhrem AA. [Primary structure of hepatoredoxin from bovine liver mitochondria]. Bioorg Khim. 1986;12(9):1286‐1289. 3778538

[77] Okamura T, John ME, Zuber MX, Simpson ER, Waterman MR. Molecular cloning and amino acid sequence of the precursor form of bovine adrenodoxin: evidence for a previously unidentified COOH-terminal peptide. Proc Natl Acad Sci U S A. 1985;82(17):5705‐5709. 2994043 10.1073/pnas.82.17.5705 PMC390620

[78] Picado-Leonard J, Voutilainen R, Kao LC, Chung BC, Strauss JF, 3rd, Miller WL. Human adrenodoxin: cloning of three cDNAs and cycloheximide enhancement in JEG-3 cells. J Biol Chem. 1988;263(7):3240‐3244. 3343244

[79] Mittal S, Zhu YZ, Vickery LE. Molecular cloning and sequence analysis of human placental ferredoxin. Arch Biochem Biophys. 1988;264(2):383‐391. 2969697 10.1016/0003-9861(88)90303-7

[80] Chang CY, Wu DA, Lai CC, Miller WL, Chung BC. Cloning and structure of the human adrenodoxin gene. DNA. 1988;7(9):609‐615. 3229285 10.1089/dna.1988.7.609

[81] Morel Y, Picado-Leonard J, Wu DA, et al Assignment of the functional gene for human adrenodoxin to chromosome 11q13----qter and of adrenodoxin pseudogenes to chromosome 20cen----q13.1. Am J Hum Genet. 1988;43(1):52‐59. 2837084 PMC1715281

[82] Griffin A, Parajes S, Weger M, et al Ferredoxin 1b (Fdx1b) is the essential mitochondrial redox partner for cortisol biosynthesis in zebrafish. Endocrinology. 2016;157(3):1122‐1134. 26650568 10.1210/en.2015-1480 PMC4769370

[83] Oakes JA, Li N, Wistow BRC, et al Ferredoxin 1b deficiency leads to testis disorganization, impaired spermatogenesis, and feminization in zebrafish. Endocrinology. 2019;160(10):2401‐2416. 31322700 10.1210/en.2019-00068

[84] Sheftel AD, Stehling O, Pierik AJ, et al Humans possess two mitochondrial ferredoxins, Fdx1 and Fdx2, with distinct roles in steroidogenesis, heme, and Fe/S cluster biosynthesis. Proc Natl Acad Sci U S A. 2010;107(26):11775‐11780. 20547883 10.1073/pnas.1004250107 PMC2900682

[85] Qi W, Li J, Cowan JA. Human ferredoxin-2 displays a unique conformational change. Dalton Trans. 2013;42(9):3088‐3091. 23208207 10.1039/c2dt32018e PMC3622203

[86] Shi Y, Ghosh M, Kovtunovych G, Crooks DR, Rouault TA. Both human ferredoxins 1 and 2 and ferredoxin reductase are important for iron-sulfur cluster biogenesis. Biochim Biophys Acta. 2012;1823(2):484‐492. 22101253 10.1016/j.bbamcr.2011.11.002 PMC3546607

[87] Rouault TA . Mammalian iron-sulphur proteins: novel insights into biogenesis and function. Nat Rev Mol Cell Biol. 2015;16(1):45‐55. 25425402 10.1038/nrm3909

[88] Stehling O, Lill R. The role of mitochondria in cellular iron-sulfur protein biogenesis: mechanisms, connected processes, and diseases. Cold Spring Harb Perspect Biol. 2013;5(8):a011312. 23906713 10.1101/cshperspect.a011312 PMC3721283

[89] Isaya G . Mitochondrial iron-sulfur cluster dysfunction in neurodegenerative disease. Front Pharmacol. 2014;5:29. 24624085 10.3389/fphar.2014.00029 PMC3939683

[90] Spiegel R, Saada A, Halvardson J, et al Deleterious mutation in FDX1L gene is associated with a novel mitochondrial muscle myopathy. Eur J Hum Genet. 2014;22(7):902‐906. 24281368 10.1038/ejhg.2013.269 PMC4060119

[91] Gurgel-Giannetti J, Lynch DS, Paiva ARB, et al A novel complex neurological phenotype due to a homozygous mutation in FDX2. Brain. 2018;141(8):2289‐2298. 30010796 10.1093/brain/awy172 PMC6061701

[92] Brentano ST, Black SM, Lin D, Miller WL. cAMP post-transcriptionally diminishes the abundance of adrenodoxin reductase mRNA. Proc Natl Acad Sci U S A. 1992;89(9):4099‐4103. 1315050 10.1073/pnas.89.9.4099 PMC525640

[93] Freeman MR, Dobritsa A, Gaines P, Segraves WA, Carlson JR. The dare gene: steroid hormone production, olfactory behavior, and neural degeneration in Drosophila. Development. 1999;126(20):4591‐4602. 10498693 10.1242/dev.126.20.4591

[94] Buszczak M, Freeman MR, Carlson JR, Bender M, Cooley L, Segraves WA. Ecdysone response genes govern egg chamber development during mid-oogenesis in Drosophila. Development. 1999;126(20):4581‐4589. 10498692 10.1242/dev.126.20.4581

[95] Gilbert LI . Halloween genes encode P450 enzymes that mediate steroid hormone biosynthesis in Drosophila melanogaster. Mol Cell Endocrinol. 2004;215(1–2):1‐10. 15026169 10.1016/j.mce.2003.11.003

[96] Huang X, Warren JT, Gilbert LI. New players in the regulation of ecdysone biosynthesis. J Genet Genomics. 2008;35(1):1‐10. 18222403 10.1016/S1673-8527(08)60001-6

[97] Moulos P, Alexandratos A, Nellas I, Dedos SG. Refining a steroidogenic model: an analysis of RNA-Seq datasets from insect prothoracic glands. BMC Genomics. 2018;19(1):537. 30005604 10.1186/s12864-018-4896-2 PMC6045881

[98] Fairfield H, Srivastava A, Ananda G, et al Exome sequencing reveals pathogenic mutations in 91 strains of mice with Mendelian disorders. Genome Res. 2015;25(7):948‐957. 25917818 10.1101/gr.186882.114 PMC4484392

[99] Peng Y, Shinde DN, Valencia CA, et al Biallelic mutations in the ferredoxin reductase gene cause novel mitochondriopathy with optic atrophy. Hum Mol Genet. 2017;26(24):4937‐4950. 29040572 10.1093/hmg/ddx377 PMC5886230

[100] Paul A, Drecourt A, Petit F, et al FDXR mutations cause sensorial neuropathies and expand the spectrum of mitochondrial Fe-S-synthesis diseases. Am J Hum Genet. 2017;101(4):630‐637. 28965846 10.1016/j.ajhg.2017.09.007 PMC5630197

[101] Slone J, Peng Y, Chamberlin A, et al Biallelic mutations in FDXR cause neurodegeneration associated with inflammation. J Hum Genet. 2018;63(12):1211‐1222. 30250212 10.1038/s10038-018-0515-y PMC6451867

[102] Slone JD, Yang L, Peng Y, et al Integrated analysis of the molecular pathogenesis of FDXR-associated disease. Cell Death Dis. 2020;11(6):423. 32499495 10.1038/s41419-020-2637-3 PMC7272433

[103] Stenton SL, Piekutowska-Abramczuk D, Kulterer L, et al Expanding the clinical and genetic spectrum of FDXR deficiency by functional validation of variants of uncertain significance. Hum Mutat. 2021;42(3):310‐319. 33348459 10.1002/humu.24160

[104] Jurkute N, Shanmugarajah PD, Hadjivassiliou M, et al Expanding the FDXR-associated disease phenotype: retinal dystrophy is a recurrent ocular feature. Invest Ophthalmol Vis Sci. 2021;62(6):2. 10.1167/iovs.62.6.2 PMC8107637 33938912

[105] Song SJ, Hong Y, Xu K, Zhang C. Novel biallelic compound heterozygous mutations in FDXR cause optic atrophy in a young female patient: a case report. Int J Ophthalmol. 2021;14(11):1796‐1798. 34804873 10.18240/ijo.2021.11.22 PMC8569559

[106] Yang C, Zhang Y, Li J, et al Report of a case with ferredoxin reductase (FDXR) gene variants in a Chinese boy exhibiting hearing loss, visual impairment, and motor retardation. Int J Dev Neurosci. 2021;81(4):364‐369. 33742450 10.1002/jdn.10104

[107] Yi S, Zheng Y, Yi Z, et al FDXR-associated oculopathy: congenital amaurosis and early-onset severe retinal dystrophy as common presenting features in a Chinese population. Genes (Basel). 2023;14(4):952. 37107710 10.3390/genes14040952 PMC10137360

[108] Masnada S, Previtali R, Erba P, et al FDXR-associated disease: a challenging differential diagnosis with inflammatory peripheral neuropathy. Neurol Sci. 2023;44(9):3037‐3043. 37046037 10.1007/s10072-023-06790-0 PMC10096094

[109] Rocatcher A, Desquiret-Dumas V, Charif M, et al The top 10 most frequently involved genes in hereditary optic neuropathies in 2186 probands. Brain. 2023;146(2):455‐460. 36317462 10.1093/brain/awac395

[110] Campbell T, Slone J, Metzger H, et al Clinical study of FDXR-related mitochondriopathy: genotype-phenotype correlation and proposal of ancestry-based carrier screening in the Mexican population. Genet Med Open. 2024;2:100841. 39669623 10.1016/j.gimo.2023.100841 PMC11613914

[111] Pignatti E, Slone J, Gomez-Cano MA, et al FDXR variants cause adrenal insufficiency and atypical sexual development. JCI Insight. 2024;9(14):e179071. in press. 38885337 10.1172/jci.insight.179071 PMC11383170

[112] Nordenstrom A, Falhammar H. Diagnosis and management of the patient with non-classic CAH due to 21-hydroxylase deficiency. Eur J Endocrinol. 2019;180(3):R127‐RR45. 30566904 10.1530/EJE-18-0712

[113] Baker BY, Lin L, Kim CJ, et al Nonclassic congenital lipoid adrenal hyperplasia: a new disorder of the steroidogenic acute regulatory protein with very late presentation and normal male genitalia. J Clin Endocrinol Metab. 2006;91(12):4781‐4785. 16968793 10.1210/jc.2006-1565 PMC1865081

[114] Sahakitrungruang T, Tee MK, Blackett PR, Miller WL. Partial defect in the cholesterol side-chain cleavage enzyme P450scc (CYP11A1) resembling nonclassic congenital lipoid adrenal hyperplasia. J Clin Endocrinol Metab. 2011;96(3):792‐798. 21159840 10.1210/jc.2010-1828 PMC3047228

[115] Fukami M, Hasegawa T, Horikawa R, et al Cytochrome P450 oxidoreductase deficiency in three patients initially regarded as having 21-hydroxylase deficiency and/or aromatase deficiency: diagnostic value of urine steroid hormone analysis. Pediatr Res. 2006;59(2):276‐280. 16439592 10.1203/01.pdr.0000195825.31504.28

